# Growth, productivity and profitability of potato (*Solanum tuberosum* L.) as influenced by nitrogen fertilizer and intra-row spacing in Ethiopia highlands

**DOI:** 10.1038/s41598-026-43518-4

**Published:** 2026-03-16

**Authors:** Ketemaw Mebrie, Baye Berihun, Daniel Asnake, Sefinew Shibabaw

**Affiliations:** 1 Department of Horticulture, Kon Technical & Vocational Education College, Amhara Region, Ethiopia; 2 Department of Horticulture, College of Agriculture and Environmental Sciences, Bahir Dar University, P.O. Box. 5501, Bahir Dar, Ethiopia

**Keywords:** Belete variety, Fertilizer rates, Intra-row spacing, Potato, Seed tuber size, Ecology, Ecology, Plant sciences

## Abstract

Nitrogen fertilizer and intra-row spacing are critical agronomic practices influencing potato (Solanum tuberosum L.) production. In Ethiopia, smallholder farmers often apply nitrogen and manage plant spacing without evidence-based guidelines, resulting in low productivity. A field experiment was conducted during the 2023 main rain fall season on a farmer’s field in the Ethiopian highlands to assess the growth and seed tuber yield of the potato variety ‘Belete’ under different nitrogen rates and intra-row spacings. The study evaluated four nitrogen levels (0, 55, 110, and 165 kg N ha⁻¹) and three intra-row spacings (20, 30, and 40 cm) in a factorial randomized complete block design with three replications. Interaction effects of nitrogen and spacing significantly affected days to 50% flowering, stem number, yields of very small, small, and large-sized tubers, average tuber weight, marketable yield, and total tuber yield. The highest marketable tuber yield (41.38 t ha⁻¹) was recorded with 110 kg N ha⁻¹ and 20 cm spacing. However, partial budget analysis revealed that the combination of 110 kg N ha⁻¹ and 30 cm spacing provided the highest net benefit (236,614 ETB ha⁻¹) and marginal rate of return (12,692.11%). These results underscore the need to optimize nitrogen application and plant spacing for enhanced seed tuber productivity and economic returns. Therefore, applying 110 kg N ha⁻¹ with 30 cm intra-row spacing is recommended for profitable potato production, improving income and food security for smallholder farmers in the study area and similar agro-ecologies.

## Introduction

Potato (*Solanum tuberosum* L.) ranks as the fourth most important food crop in the world after rice, wheat, and maize in terms of yield, accounting for approximately 45% of the global production of all tuber crops. It also ranks eighth globally in terms of the area under cultivation^1^. Potato is among the most productive and widely cultivated food crops, serving as both a staple food and a major source of income for many communities worldwide^2^. Globally, annual potato production is estimated at 386,750,000 tons, cultivated on about 16,800,000 hectares. In Africa, production reaches approximately 34,266,908 tons from 2,200,000 hectares^3^. In Ethiopia, the total production stands at around 991,937.48 tons, grown on 68,072.94 hectares of land^4^. Despite the country’s favorable agro-ecological conditions and the crop’s popularity, the national average yield remains low at 14.57 t ha⁻¹ significantly below the global average of 23.02 t ha⁻¹ and much lower than yields in other African countries such as South Africa (45.57 t ha⁻¹), Egypt (38.96 t ha⁻¹), and Algeria (29.10 t ha⁻¹)^3^. Several studies have identified the major factors contributing to the low productivity of potato in Ethiopia. These include the use of inappropriate agronomic practices such as suboptimal fertilizer rates and types^5^, inadequate plant population management^6^, limited availability of quality seed tubers, declining soil fertility, moisture stress, and the prevalence of pests and diseases^7,8^.

Potato is a nutrient-demanding crop, requiring high levels of both macro- and micro-nutrients due to its shallow and poorly developed root system^9^. In Ethiopia, however, most farmers continue to rely on blanket fertilizer recommendations typically 165 kg ha⁻¹ of urea and 195 kg ha⁻¹ of DAP without considering variations in soil fertility, environmental conditions, or the specific nutrient requirements of different potato varieties^10^. Numerous studies have demonstrated that nitrogen (N) plays a crucial role in improving total tuber yield and tuber size in potatoes^11^. Alemayehu et al.^12^ found that nitrogen fertilization significantly increased the proportion of large-sized tubers, while reducing the percentages of medium and small tubers. Nitrogen levels also influence crop maturity: higher nitrogen availability tends to delay maturity by promoting prolonged vegetative growth and excessive haulm development, whereas limited nitrogen favors earlier tuber initiation due to reduced vegetative growth^13^. Similarly, Zabihi^14^ reported that increased nitrogen application generally enhances tuber number. However, excessive nitrogen application can reduce yield and tuber quality, underscoring the importance of applying nitrogen at optimal rates. Excessive nitrogen use also leads to nitrate (NO₃⁻) leaching into groundwater, eutrophication of surface waters, soil acidification^15^, and increased emissions of nitrous oxide (N₂O), a potent greenhouse gas, thereby degrading ecosystems and contributing to climate change^16^.

Another critical consideration in determining plant population and optimal spacing in potato production is the high seed rate required, which can account for up to 50% of the total production cost^17^. Plant density is a key agronomic factor, as it directly influences seed cost, crop development, yield, and tuber quality^18^. In Ethiopia, a blanket recommendation of 60 cm between rows and 30 cm between plants is applied for seed potato production across all varieties, including Belete a variety known for producing large seed tubers^19^. Intra-row spacing, in particular, has been shown to significantly impact seed potato yield. Rahemi et al.^20^ reported that a 20 cm intra-row spacing increased yield by 36.39% compared to 30 cm spacing. However, several studies have also indicated that closer spacing tends to reduce average tuber weight compared to wider spacing^21,22^. These variations in response to plant spacing are often influenced by local factors such as soil fertility, moisture availability, and the growth characteristics of the specific potato variety^23^. Given these considerations, the present study was initiated with the aim of enhancing smallholder farmers’ income and improving food security by identifying the optimal nitrogen fertilizer rate and intra-row spacing for the economical production of potato.

## Materials and methods

### Description of the study area

The study was conducted in Wadla district of North Wollo Zone, Amhara Regional State, Ethiopia. The crop is the major income source of smallholder farmers in North Wollo Zone. The area was therefore purposively selected. Geographically the area is located between 38° 44’ 368’’ E longitude and 11° 49’ 401’’ N latitude and with an altitude of 2876 m above sea level. According to Bahir Dar Meteorology Service Center report (unpublished), the annual temperature and rainfall are 5–25 °C and 500–1200 mm, respectively. The common dominant soil type of the area is Cambisols.

### Description of experimental material

For this experiment, the improved potato variety Belete, sourced from the Sirinka Agricultural Research Center, was used. Belete was originally released in 2009 by the Holetta Agricultural Research Centre (HARC)^19^. The variety grows best at altitudes between 1,600 and 2,800 m above sea level and under rainfall conditions ranging from 750 to 1,000 mm. It reaches maturity in about 110 days^24,25^. It is one of the most widely cultivated potato varieties in Ethiopia, favored by farmers for its high yield potential, strong market demand, and consumer-preferred quality. It exhibits relatively better tolerance to late blight compared to many other improved varieties and local cultivars^2^. This improved cultivar is particularly well-suited to highland areas and is recognized for its large tuber size, relatively early maturity, desirable tuber quality traits, and adaptability under diverse management practices. On average, it produces larger tubers with good specific gravity^25,26^.

### Experimental treatment, design and procedures

Treatments consisted of four nitrogen levels (0, 55, 110, and 165 kg ha⁻¹) applied in the form of urea, combined with three intra-row spacings (20, 30, and 40 cm). The experiment was laid out in a factorial arrangement using a Randomized Complete Block Design (RCBD) with three replications. Each experimental plot covered an area of 9 m² (3 m × 3 m) and consisted of five rows with 60 cm spacing between rows. Depending on the intra-row spacing, the number of plants per row was 15 at 20 cm, 10 at 30 cm, and 8 at 40 cm. Consequently, the net plot size varied with the spacing treatment. To minimize interference, plots were separated by 1 m, while blocks were spaced 1.5 m apart.

### Management of experimental plant

The experimental field was ploughed three times with oxen to a depth of 25–30 cm, and plots were prepared following standard procedures. Medium-sized, healthy, and well-sprouted Belete seed tubers (weighing 39–75 g) were planted at a depth of 8–10 cm^26,27^. NPS fertilizer was applied at the nationally recommended rate of 196 kg ha⁻¹. Nitrogen, supplied in the form of urea, was applied in three split doses: one-third at planting, one-third at crop emergence, and the final one-third at tuber initiation^19^. All other management practices—including weeding, cultivation, and earthing-up—were carried out uniformly across all plots according to recommended practices and as needed^28,29^.

### Soil sampling and analysis

Before planting, soil samples were collected from a depth of 0–30 cm using a zigzag sampling pattern across the experimental field. A total of 20 sub-samples were taken, thoroughly mixed to form a composite sample, and then analyzed in the laboratory for key physical and chemical properties using standard procedures. Soil pH was determined with a glass electrode pH meter at a 1:2.5 soil-to-water ratio. Organic carbon (OC) was measured using the wet oxidation method^30^, while total nitrogen (TN) was analyzed using the Kjeldahl method^31^ and the available Phosphorus (P) was analyzed using the Olsen method^32^. The results of the soil’s physical and chemical characteristics are summarized in Table 1.

**Table 1:** Major soil characteristics of the experimental site.

Soil properties	Value	Rating	Reference
Soil pH	5.9	Moderately acidic (5.6–6.5)	^30^
Organic carbon (%)	1.248	Low (2–4%)	^30^
Total N (%)	0.315	Medium (0.21–0.5%)	^31^
Available P (mg/kg)	low	Low (15–30)	^32^
CEC (Cmol/kg)	48.535		
EC (Ds/m)	Salt free		
Organic Matter (%)	4.71	Very high	
Bulk Density (g cm-3)	1.13		

### Data collection

#### Phenological parameters

Days to 50% emergence were recorded as the number of days from planting until half of the tubers in a plot had emerged^25^. Similarly, days to 50% flowering were determined by counting the days from planting until 50% of the plants in each plot had started flowering. Days to 90% maturity were measured as the time from planting to the stage when about 90% of the plants in a plot showed leaf and stem senescence^33^.

#### Growth parameter

Plant height was measured at 50% flowering by taken five plants at random from the net plot area and recording their height from the soil surface to the tip of the main stem using a ruler. The average of these measurements was then used for analysis^25^. In the same way, stem number per hill was recorded at 50% flowering by counting the stems of five randomly selected hills within the net plot area and calculating the mean. Only stems that emerged directly from the mother tuber were considered as primary stems^25^.

#### Yield and yield related parameters

Tubers harvested from the net plot were categorized into four size classes following Lung’aho et al^27^. very small (< 25 g), small (25–38 g), medium (39–75 g), and large (> 75 g). Tubers in each category were weighed separately and converted to yield per hectare (t ha⁻¹). Average tuber weight was calculated by dividing the total tuber weight per plant by the total number of tubers harvested per plant. Marketable yield was determined as the weight of tubers ≥ 25 g that were free from mechanical damage, diseases, and insect pest attack^23,30^. These tubers were weighed using a scale balance, and the measurements were converted to t ha⁻¹. Conversely, tubers weighing < 25 g, or those that were deformed, misshapen, or rotting, were considered unmarketable^25,34^. Their weights were also recorded and expressed in t ha⁻¹. Total tuber yield was then obtained by summing the marketable and unmarketable yields^25^.

### Data analysis

All collected potato data were analyzed using Analysis of Variance (ANOVA) with the Statistical Analysis Software (SAS) version 9.2^35^. When treatment effects were found to be significant, mean comparisons were carried out using the Least Significant Difference (LSD) test at either the 1% or 5% probability level^36^.

### Economic analysis

The partial budget and marginal rate of return (MRR) were evaluated following the guidelines of CIMMYT^37^. For the MRR analysis, labor, fertilizer, and seed tubers were considered as variable costs. Gross benefits for each treatment were calculated using the adjusted yield and the prevailing field price of the crop, while net benefits were obtained by subtracting the variable costs from the gross benefits. The marginal rate of return was then calculated by dividing the change in net benefit by the change in total treatment cost^37^.

## Results and discussion

### Phenological parameters of potato

#### Days to 50% emergence

Neither the main effects nor the interaction of nitrogen fertilizer and intra-row spacing had a significant impact on days to 50% emergence of potato plants. This suggests that plant emergence is largely governed by the crop’s genetic traits rather than by nitrogen application or plant spacing. These findings are consistent with those of Elfinesh^38^, who reported that crop phenological characteristics are primarily determined by the inherent characteristics of the variety.

Planting schedules strongly affect potato phenology, including emergence, flowering, and tuber initiation. Optimal planting dates align growth with favorable environmental conditions, shortening maturity and maximizing tuber yield, while early or late planting can reduce biomass and marketable yield^39^. Similarly, crop rotation improves vegetative growth, photosynthetic efficiency, and tuber yield compared to continuous potato cropping^40^.

#### Days to 50% flowering

Nitrogen fertilizer (*P* < 0.001) and intra-row spacing (*P* < 0.01) both had a significant effect on the days to flowering of potato plants, and their interaction was also significant (*P* < 0.05). The longest time to flowering (82.33 days) was observed in plants receiving 165 kg N ha⁻¹ at the widest intra-row spacing, while the shortest time (65.33 days) occurred in the control treatment with narrow spacing (Table 2). This delay in flowering with higher nitrogen levels may be attributed to the fact that nutrient deficiency and limited aeration in unfertilized plots create stress during active growth, leading to earlier flowering. Conversely, increasing nitrogen application promotes vegetative growth and delays flowering, as reported by previous studies^41,42^.

Similarly, increasing intra-row spacing significantly delayed flowering. Potato plants grown in narrow spacing may experience higher competition for nutrients, which can induce stress and accelerate flowering, while wider spacing allows better light penetration and nutrient availability, supporting prolonged vegetative growth. These observations align with previous findings^34,41^.

**Table 2:** Interaction effects of N fertilizer and intra-row spacing on days to flowering of potato.

*N* fertilizer (kg ha^− 1^)	Intra-row spacing (cm)		
	20	30	40
0	65.33^g^	69.00^f^	69.67^f^
55	75.00^e^	76.33c^de^	76.00^de^
110	80.67^ab^	76.33^cde^	78.00^cd^
165	78.67^bc^	81.00^ab^	82.33^a^
p-value			***
CV (%)			1.89
SE±			0.23

*** = significant at *p* < 0.001; means followed by the same letter (s) are not significantly different; CV = coefficient of variation; SE = standard error.

#### Days to 90% maturity

Plant maturity was highly significantly (*P* < 0.001) affected by the main effects of nitrogen fertilizer and intra-row spacing, while their interaction had no significant influence. Potato plants grown without nitrogen matured earlier (101.0 days) compared to those receiving 165 kg N ha⁻¹, which took the longest to reach maturity (109.44 days) (Table 3). This delay in maturity with higher nitrogen levels is attributed to the nutrient’s role in promoting vegetative growth, which prolongs the growing period and delays tuber formation. These findings are consistent with Tigist and Asrat^41^, who reported that nitrogen strongly influences ground cover throughout the season and extends the growing period, resulting in delayed maturity. Excessive nitrogen can also reduce the allocation of dry matter to tubers, further delaying physiological maturity^42^. Overall, nitrogen fertilization delayed flowering and extended the time required for potato plants to reach full maturity.

The earliest 90% maturity (104.25 days) was recorded in potato plants grown at the closest intra-row spacing of 20 cm. This may be attributed to the intense competition among plants at narrower spacing, which can deplete available nutrients, causing stress and leading to earlier maturity. This result is in accordance with Boto et al^43^, who reported that higher planting densities promote early tuber development and accelerate maturity in potatoes.

**Table 3:** Main effects of N fertilizer and intra-row spacing on phenological parameters of potato.

*N* fertilizer (kg ha^−1^)	Days to 50% emergence	Days to 50% flowering	Days to 90% maturity
0	13.78	68.11^d^	101.00^c^
55	13.89	75.78^c^	105.89^b^
110	13.89	78.33^b^	107.11^b^
165	14.44	80.67^a^	109.44^a^
p-value	ns	***	***
Intra-row spacing			
20	14.33	74.92^b^	104.25^b^
30	13.67	75.67^ab^	106.33^a^
40	14	76.58^a^	107.00^a^
p-value	ns	*	***
CV (%)	10.42	1.89	1.36
SE±	0.24	0.23	0.24

*** = significant at *p* < 0.001; * = significant at *p* < 0.05; ns = non-significant; means followed by the same letter (s) within a column are not significantly different; CV = coefficient of variation; SE = standard error.

### Growth parameters of potato

#### Plant height

Plant height of potato was significantly influenced (*P* < 0.001) by both nitrogen fertilizer and intra-row spacing, although their interaction had no significant effect. The tallest plants (80.99 cm) were observed with the application of 165 kg N ha⁻¹, while the shortest plants (44.71 cm) were recorded in the unfertilized control (Table 4). This increase in plant height with nitrogen application is likely due to improved soil nutrient availability and enhanced soil aeration, which support better vegetative growth. Similar results were reported by Fayera^42^ and Khalafalla^44^, who found that increasing nitrogen levels promoted taller potato plants.

Regarding intra-row spacing, the tallest plants (67.89 cm) were observed at the widest spacing of 40 cm, while the shortest plants (58.51 cm) were recorded at the closest spacing of 20 cm (Table 4). The greater plant height at wider spacing is likely due to reduced competition for sunlight, allowing individual plants to grow more vigorously. These findings are consistent with Tigist and Asrat^41^and Zamil et al^45^, who reported that wider intra-row spacing, which reduces plant density, promotes taller potato plants.

Denser planting and high nitrogen rates can increase labor for weeding, hilling, and harvesting, reduce canopy air circulation favoring foliar diseases (e.g., late blight), promote lush growth exacerbating disease susceptibility, and raise risks of N leaching, runoff pollution, inefficient N use, and GHG emissions. Integrated management is essential to optimize productivity while minimizing environmental impacts. Excessive N leads to excessive vegetative growth, delayed tuber maturation, increased disease sensitivity, nutrient leaching, and higher GHG emissions, while optimal density-N combinations improve yield and quality without these drawbacks^46–48^.

**Table 4:** Plant height of potato as influenced by the main effects of N fertilizer and intra-row spacing.

*N* fertilizer (kg ha^− 1^)	Plant height (cm)
0	44.71^d^
55	55.76^c^
110	71.76^b^
165	80.99^a^
p-value	***
Intra-row spacing	
20	58.51^c^
30	63.51^b^
40	67.89^a^
p-value	***
CV (%)	4.29
SE±	0.08

*** = significant at *p* < 0.001; means followed by the same letter (s) within the same column are not significantly different; CV = coefficient of variation; SE = standard error.

#### Stem number

Stem number is a critical parameter contributing to the yield potential of potato. In this study, it was significantly (*P* < 0.001) influenced by both the main effects and the interaction of nitrogen fertilizer and intra-row spacing. The highest number of stems per hill (8.27) was recorded in plants receiving 165 kg N ha⁻¹ at 40 cm intra-row spacing, while the lowest (3.66) occurred in the unfertilized control at the closest spacing (Table 5). This increase in stem number with nitrogen application is likely due to enhanced growth and development, which promotes the formation of more stems. These findings align with Tigist and Asrat^41^, who reported that nitrogen fertilization significantly increases the number of stems per plant.

Similarly, the higher stem number observed at 40 cm spacing may be attributed to reduced competition among plants for growth resources such as light, moisture, and soil nutrients. Berga et al^49^ also reported that intra-row spacing affects stem development. Overall, the results indicate that both fertilizer application and plant density play important roles in determining the number of stems per potato plant.

**Table 5:** Stem number per hill of potato as influenced by interaction effect of N fertilizer and intra-row spacing.

*N* fertilizer (kg ha^− 1^)	Intra-row spacing (cm)		
	20	30	40
0	3.93^g^	4.87^f^	4.27^g^
55	5.40^e^	5.87^de^	6.00^d^
110	6.57^c^	6.80^c^	5.6d^e^
165	7.47^b^	7.97^a^	8.27^a^
p-value			***
CV (%)			4.77
SE±			0.01

*** = significant at *p* < 0.001; treatment means followed by the same letter (s) are not significantly different; CV = coefficient of variation; SE= standard error.

### Yield and yield related parameters of potato

#### Very small-sized (< 25 g) tuber yield

**Fig. 1:**
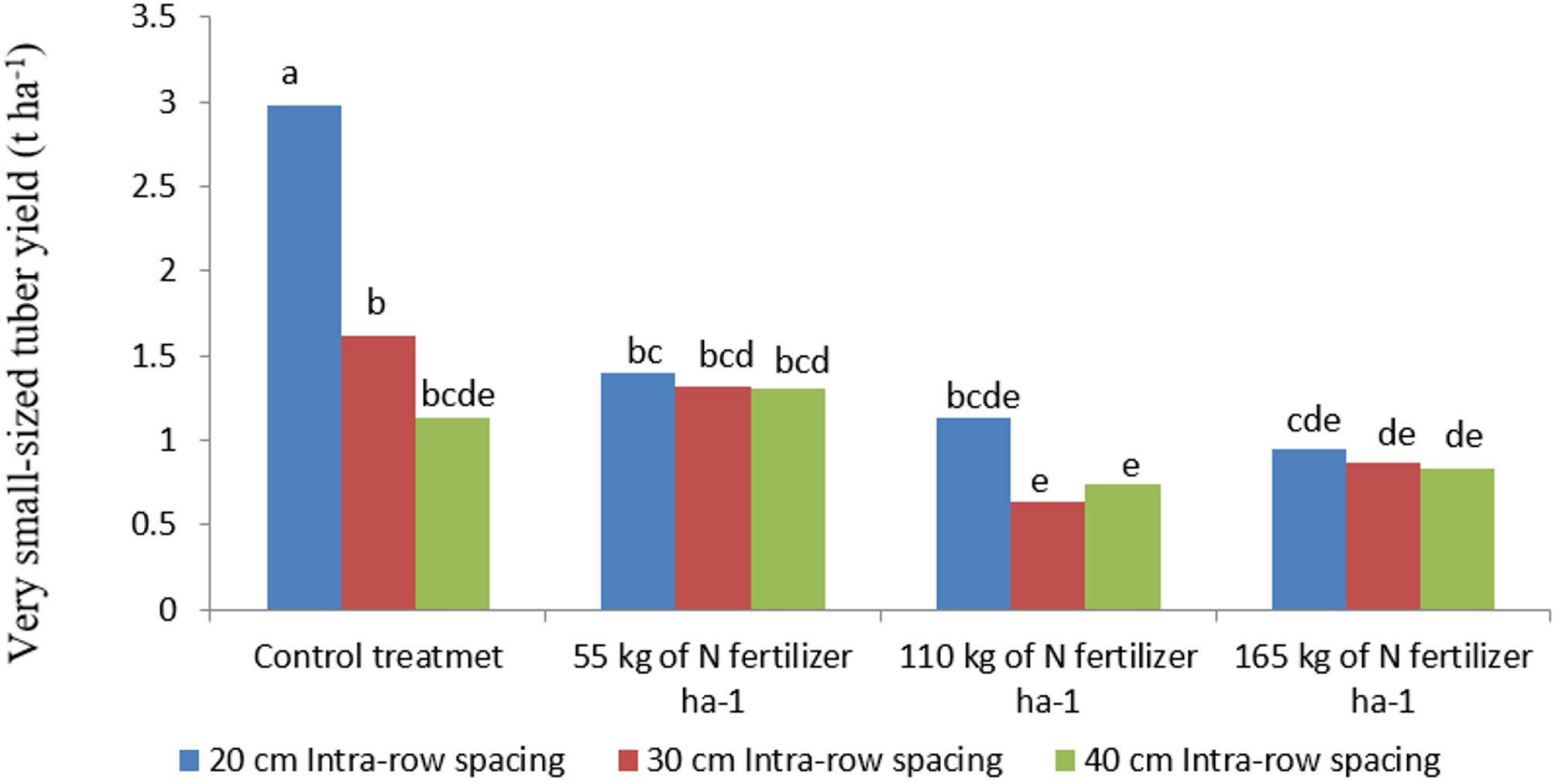
Interaction effect of N fertilizer and intra-row spacing on very small-sized tuber yield of potato. Note: Mean values with the same letter/s are statistically similar at *P* < 0.001.

Nitrogen fertilizer and intra-row spacing significantly influenced the yield of very small-sized potato tubers both in their main effects (*P* < 0.01) and in their interaction effects (*P* < 0.001). The highest yield of very small-sized tubers (2.98 t ha⁻¹) was recorded under the control treatment combined with closer intra-row spacing, whereas the lowest yield (0.64 t ha⁻¹) was obtained with 110 kg N ha⁻¹ applied at 30 cm intra-row spacing, as illustrated in Fig. 1. These findings are consistent with Iritani^50^, who reported that increasing nitrogen application enhances both total potato yield and tuber size.

The observed differences in tuber size across intra-row spacings are likely due to the higher plant population density at narrower spacings, which increases competition for nutrients and other growth resources, ultimately resulting in a higher proportion of very small tubers. Overall, decreasing intra-row spacing from wider to narrower intervals tended to increase the proportion of very small-sized tubers. Tesfaye et al^51^ similarly reported that narrow intra-row spacing produced the highest number of small-sized tubers. Likewise, Bikila et al^52^ found that narrower spacing increased the number of undersized tubers, leading to potential economic losses for farmers, since medium-sized tubers are preferred for both seed purposes and higher market value.

#### Small-sized (25–38 g) tuber yield

**Fig. 2:**
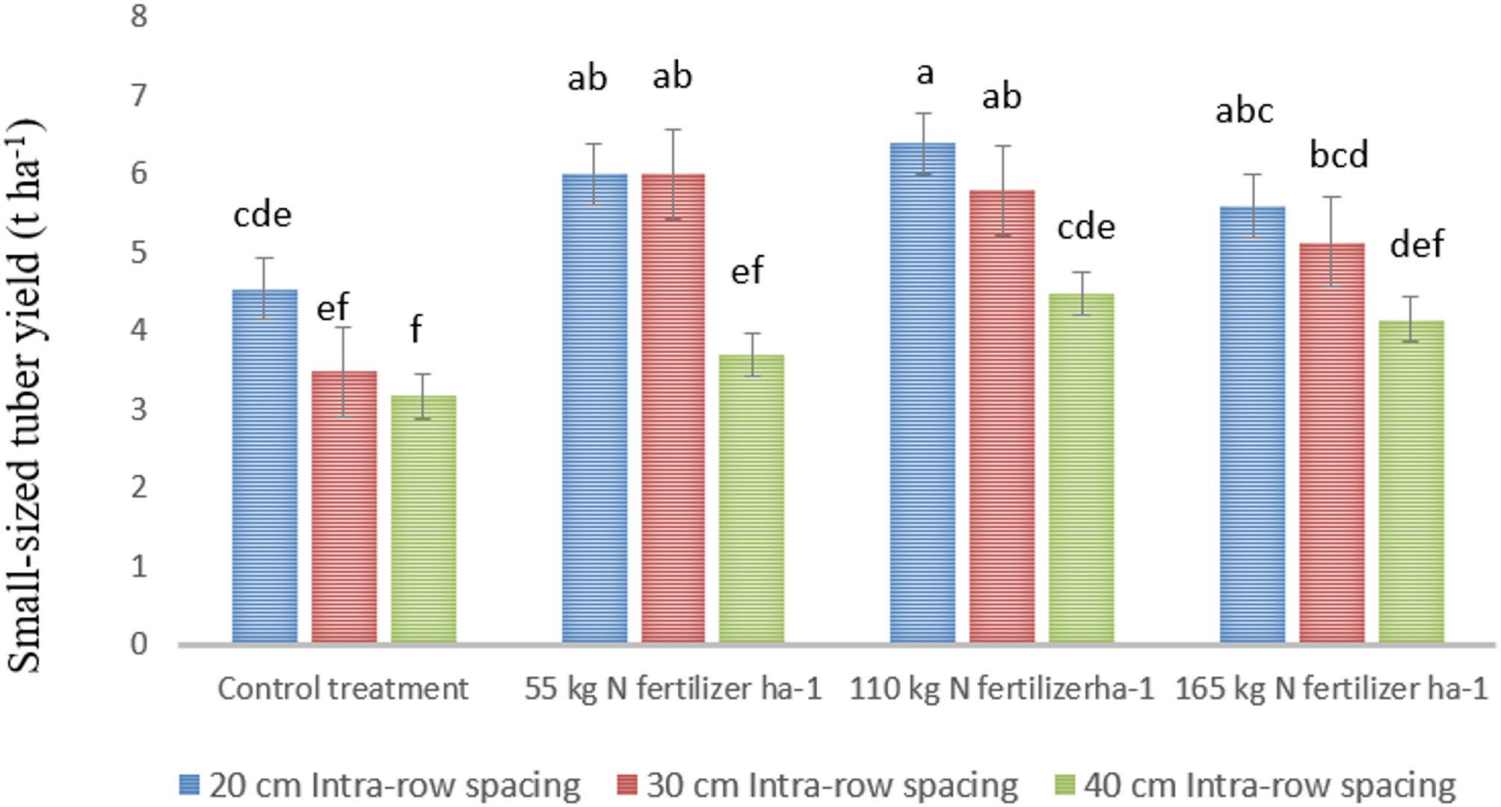
Interaction effect of N fertilizer and intra-row spacing on small-sized tuber yield of potato. Note: Mean values with the same letter/s are statistically similar at *P* < 0.001.

The yield of small-sized potato tubers was significantly (*P* < 0.01) affected by both the main effects and the interaction effects of nitrogen (N) fertilizer and intra-row spacing. The highest small-sized tuber yield (6.39 t ha⁻¹) was obtained from the combination of 110 kg N ha⁻¹ with closer intra-row spacing, whereas the lowest yield (3.17 t ha⁻¹) was recorded under the control treatment with wider intra-row spacing, as shown in Fig. 2. These results align with Feyera^42^, who reported that nitrogen application increased the yield of all tuber grades and slightly enhanced the weight of individual tubers.

Closer plant spacing resulted in higher yields of small-sized seed tubers compared to wider spacing. This is likely due to increased competition for nutrients and other growth resources among plants at closer spacing, which promotes the production of smaller tubers. This observation agrees with Zebenay^53^, who reported that the total number of tubers and the proportion of smaller seed tubers increased with closer spacing. Conversely, Mass^54^ noted that wider spacing favors the production of larger tubers but reduces the yield of very small, small, and unmarketable tubers.

#### Medium-sized (39–75 g) tuber yield

**Fig. 3:**
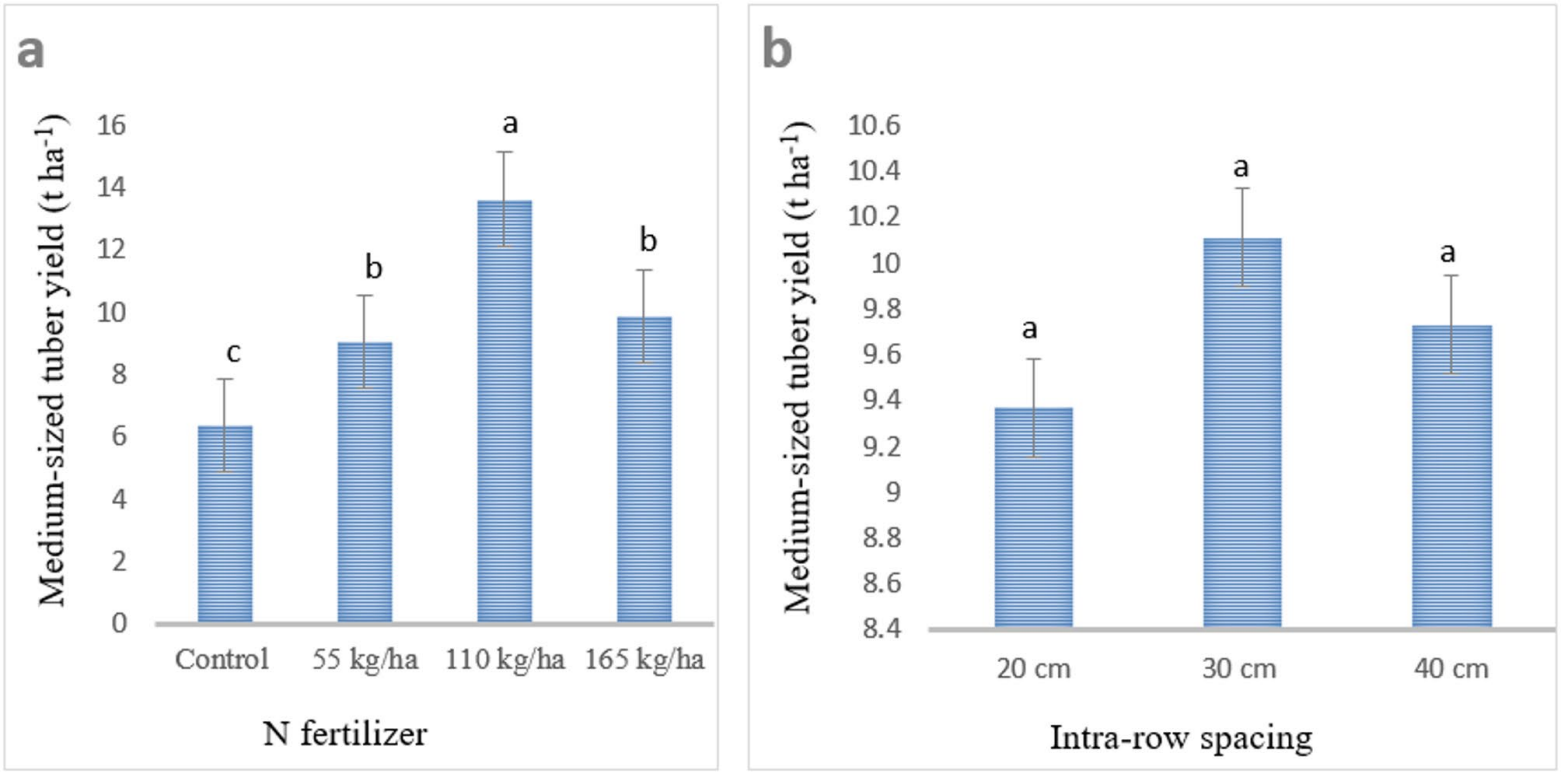
Influence of N fertilizer (**a**) and intra-row spacing; (**b**) on medium-sized tuber yield (t ha^− 1^) of potato. Note: Mean values with the same letter within the group are statistically similar at *p* < 0.001 and *p* < 0.05, respectively.

Nitrogen fertilizer and intra-row spacing significantly affected medium-sized tuber yield, with N fertilizer having a very highly significant effect (*P* < 0.001) and intra-row spacing a significant effect (*P* < 0.05). However, their interaction did not significantly influence medium-sized tuber yield. Application of 110 kg N ha⁻¹ resulted in the highest medium-sized tuber yield (13.63 t ha⁻¹), while the lowest yield (6.37 t ha⁻¹) was recorded under the control treatment, as shown in Fig. 3. These findings are consistent with Wurr^55^, who reported that nitrogen application increases the yield of medium and large tubers. This effect is likely due to enhanced growth and weight of individual tubers, which shifts tubers from smaller to medium and large size categories.

Medium-sized tuber yield was also influenced by intra-row spacing. The highest yield (10.11 t ha⁻¹) was observed at 30 cm intra-row spacing, whereas the lowest yield (9.37 t ha⁻¹) occurred at closer spacing (20 cm), as shown in Fig. 3. The reduced yield at closer spacing may be attributed to greater competition among plants for nutrients and other growth factors, limiting the development of medium-sized tubers. In contrast, wider spacing provides sufficient room for root and tuber expansion, minimizes competition, and allows for more efficient nutrient uptake and photosynthesis, resulting in higher yields of medium-sized tubers. Overall, these results indicate that intra-row spacing significantly influences potato tuber size distribution. By selecting appropriate planting densities, it is possible to produce seed potatoes or ware potatoes of the desired size to meet market and consumer demands^56^.

#### Large-sized (> 75 g) tuber yield

**Fig. 4:**
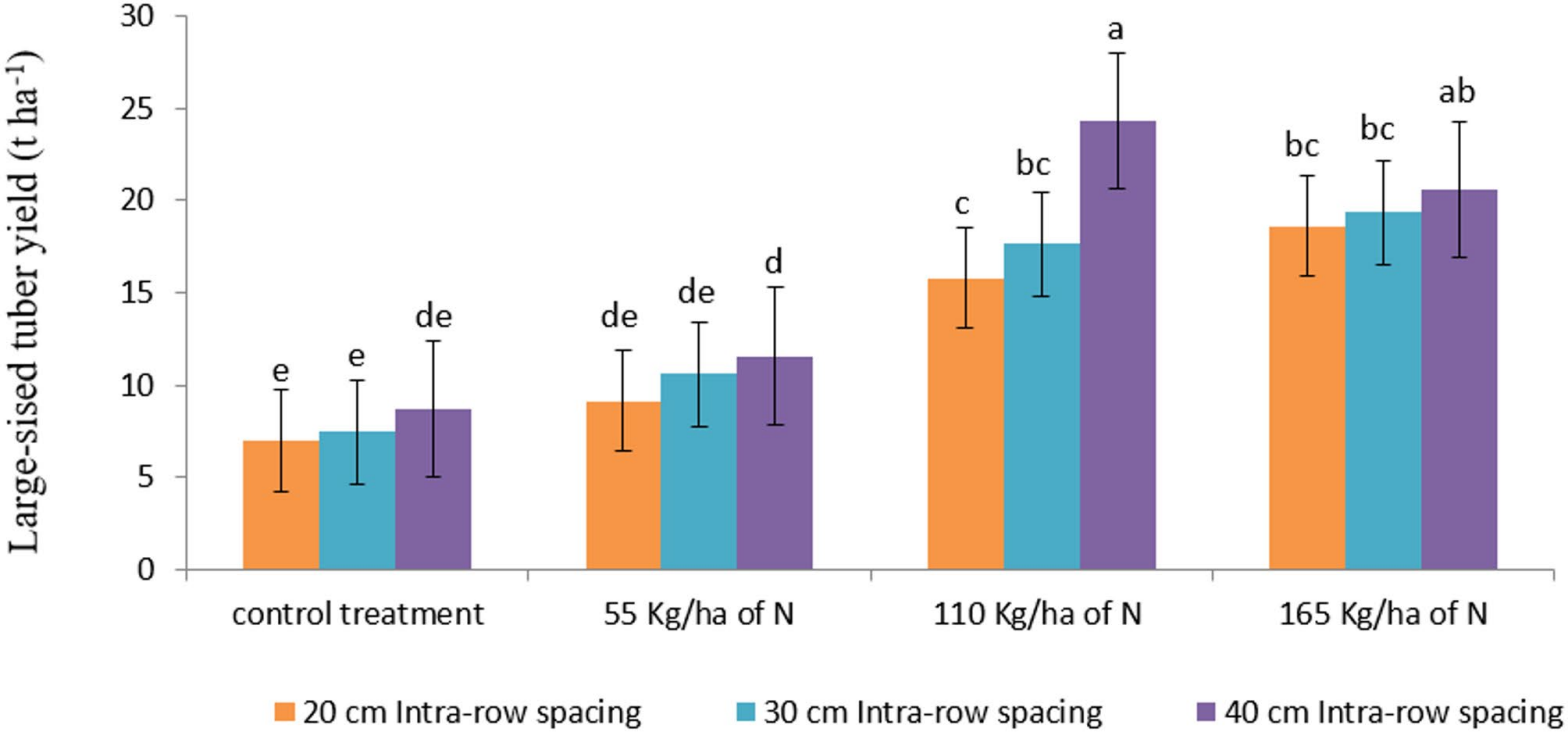
Interaction effect of N fertilizer and intra-row spacing on large-sized tuber yield of potato. Note: Mean values with the same letter/s are statistically similar at *P* < 0.001.

The analysis of variance revealed that the main effects of N fertilizer and intra-row spacing had very highly significantly (*P* < 0.001) and significantly (*P* < 0.05) influenced large-sized tuber yield, respectively. Moreover, their interaction was also found to be very highly significant (*P* < 0.001). The highest yield of large-sized tubers (24.30 t ha⁻¹) was recorded when potatoes were planted at a 40 cm intra-row spacing combined with 110 kg ha⁻¹ of N fertilizer. In contrast, the lowest yield (7.00 t ha⁻¹) was obtained from the densest planting arrangement 20 cm intra-row spacing with no nitrogen application (Fig. 4).

The increased yield of large-sized tubers at wider intra-row spacing observed in this study can be attributed to reduced plant-to-plant competition for nutrients, light, and space. With less competition, individual plants have better access to resources, which promotes vigorous growth and the development of larger tubers. These findings align with previous reports by Tesfaye et al^51^, who noted that wider intra-row spacing leads to larger tuber sizes due to improved resource availability and enhanced photosynthetic activity. Similarly, Alemayehu et al^57^ also reported that wider spacing significantly increased the yield of larger tubers in potato crops.

#### Average tuber weight per hill

The analysis of variance revealed that both the main effects and the interaction between nitrogen fertilizer and intra-row spacing had a significant influence (*P* < 0.001) on average tuber weight. The highest average tuber weight (2.63 kg per hill) was recorded in plants that received 110 kg N ha⁻¹ and were spaced at 40 cm intra-row distance. In contrast, the lowest average tuber weight (0.57 kg per hill) was observed in the control treatment with no nitrogen application and a closer spacing of 20 cm (Table 6). Across all spacing treatments, the application of 110 kg N ha⁻¹ consistently produced the highest average tuber weight compared to other fertilizer levels.

The reduction in average tuber weight at narrower intra-row spacing could likely be attributed to increased competition among plants for essential growth resources such as nutrients, light, and water. This heightened competition may limit the availability of nutrients per plant, ultimately leading to reduced tuber development and lower average tuber weight. These findings are consistent with previous studies^58–60^, which reported higher average tuber weight per hill at wider plant spacing compared to denser spacing. In general, a decrease in planting density has been shown to promote greater tuber weight per hill due to improved access to resources.

#### Marketable tuber yield

The analysis of variance indicated that marketable tuber yield was significantly affected by both nitrogen fertilizer and intra-row spacing. Specifically, N fertilizer had a very highly significant effect (*P* < 0.001), while intra-row spacing had a significant effect (*P* < 0.05). Furthermore, their interaction had a very highly significant effect (*P* < 0.001) on marketable tuber yield. Marketable tuber yield is a key agronomic trait that directly influences the profitability of potato production. In this study, the combination of 110 kg N ha⁻¹ with a closer intra-row spacing (20 cm) resulted in the highest marketable tuber yield (41.38 t ha⁻¹), whereas the lowest yield (15.46 t ha⁻¹) was recorded in the control treatment (no nitrogen) with the same closer spacing (Table 6). This significant improvement in yield with nitrogen application can be attributed to its vital role in plant growth and development, as nitrogen is a key component of chlorophyll and proteins, both essential for photosynthesis and biomass accumulation^61^.

The higher marketable tuber yield observed at closer intra-row spacing is likely due to the increased number of plants per unit area, which results in a greater number of tubers produced per hectare. Although individual tuber size may be smaller under denser spacing, the total number of marketable-sized tubers can be higher, leading to an increased overall yield. These findings are consistent with those of Sebnie et al^62^, Alemayehu et al^12^, Gebremariam^63^, and Israel et al^13^, who reported a significant increase in marketable yield with increasing nitrogen rates.

Similarly, Kasaye^56^and Harnet et al^59^ found that closer intra-row spacing (20 cm) resulted in a higher proportion of marketable tubers compared to wider spacing (35 cm). Zebenay^53^ also reported that a spacing of 20 × 50 cm produced the highest marketable tuber yield. These results highlight the importance of optimizing plant density and nutrient supply to maximize the economic return from potato cultivation.

**Table 6:** Yield components of potato as influenced by interaction effects of N fertilizer and intra-row spacing.

*N* fertilizer	Intra-row spacing (cm)	ATW (kg)	MTY (t ha^−1^)	TTY (t ha^−1^)
0	20	0.57^g^	15.46^g^	20.71^f^
	30	0.63^fg^	17.76^fg^	20.97^f^
	40	0.77^efg^	18.59^fg^	22.52^ef^
55	20	0.90^def^	19.91^fg^	20.86^f^
	30	0.93^de^	19.96^fg^	27.13^de^
	40	0.93^de^	23.18^ef^	37.47b^c^
110	20	1.40^c^	41.38^a^	33.93^c^
	30	1.73^b^	39.77^ab^	44.59^a^
	40	2.63^a^	35.25^bc^	41.95^ab^
165	20	0.58^g^	28.16^de^	23.06^ef^
	30	0.73^efg^	31.10^cd^	31.65^cd^
	40	1.17^cd^	31.88^cd^	34.39^c^
p-value		***	***	***
CV (%)		15.59	13.60	0.03
SE±		0.61	12.21	0.61

*** = significant at *p* < 0.001; means followed by the same letter (s) are not significantly different; CV = coefficient of variation; SE = standard error; ATW = average tuber weight per hill; MTY = marketable tuber yield; TTY = total tuber yield.

#### Unmarketable tuber yield

The analysis of variance showed that both nitrogen (N) fertilizer and intra-row spacing had a significant effect (*P* < 0.001) on unmarketable tuber yield of potato. However, their interaction did not significantly affect this parameter.

The highest unmarketable tuber yield (3.37 t ha⁻¹) was recorded in the control treatment (no N fertilizer), while the lowest (2.48 t ha⁻¹) was observed with the application of 110 kg N ha⁻¹. The increased unmarketable yield in the control plots could be due to severe competition among plants for limited resources such as nutrients and light, resulting in reduced photosynthetic efficiency and lower assimilate production. This, in turn, may have led to the development of smaller or misshapen tubers deemed unmarketable. These findings are in agreement with the results of Fayera^42^, who reported higher unmarketable tuber yields in potato plants grown without nitrogen application.

With respect to intra-row spacing, the highest unmarketable tuber yield (3.96 t ha⁻¹) was obtained from the closest spacing (20 cm), while the lowest (2.45 t ha⁻¹) was recorded at the widest spacing (40 cm). However, the difference in unmarketable tuber yield between the 30 cm and 40 cm spacing was not statistically significant (Table 7). Generally, higher unmarketable tuber yield at closer spacing can be attributed to increased plant density, which intensifies competition for essential growth factors such as nutrients, light, and water. This increased competition can limit individual plant development, resulting in a higher proportion of small or malformed tubers that fall outside the marketable grade. These results are consistent with the findings of Tesfa et al^60^ and Jemal^64^, who also reported that closer plant spacing resulted in higher unmarketable tuber yields compared to wider spacing.

**Table 7:** Main effects of N fertilizer and intra-row spacing on unmarketable tuber yield of potato.

*N* fertilizer (kg ha^− 1^)	Unmarketable tuber yield (t ha^− 1^)
0	3.37^a^
55	3.09^a^
110	2.48^b^
165	3.31^a^
p-value	***
Intra-row spacing	
20	3.96^a^
30	2.78^b^
40	2.45^b^
p-value	***
CV (%)	13.08
SE±	0.07

*** = significant at *p* < 0.001; means followed by the same letter (s) within the same column are not significantly different; CV=coefficient of variation; SE= standard error.

#### Total tuber yield

The analysis of variance showed that total tuber yield of potato was significantly (*P* < 0.001) affected by both the main effects of nitrogen fertilizer and intra-row spacing, as well as by their interaction. The highest total tuber yield (44.59 t ha⁻¹) was recorded from the combination of 110 kg N ha⁻¹ with 30 cm intra-row spacing. In contrast, the lowest total tuber yield (20.71 t ha⁻¹) was obtained from the control treatment (no nitrogen) at the closer spacing (20 cm) (Table 6). The increased yield with higher nitrogen application is likely due to enhanced vegetative growth, including greater stem production and a higher number of tubers, which ultimately contribute to increased yield^42^.

Although higher total yields were recorded at medium closer spacing (30 cm) under sufficient nitrogen, wider spacing generally favors the development of stronger individual plants by reducing intra-specific competition for essential resources such as nutrients, light, and moisture. In denser plant populations, competition intensifies, which may limit the nutrient availability per plant, reducing overall growth and, consequently, total tuber yield. This finding is consistent with the results reported by Arega et al^58^, who noted that excessive plant density can negatively affect yield due to increased competition.

### Partial budget analysis of potato as influenced by N fertilizer and intra-row spacing

Partial budget analysis was conducted by considering total variable costs and net benefits for each treatment. To better reflect actual farmer production conditions, the marketable yield was adjusted downward by 10%. At the time of harvest, the average farm-gate price for potatoes was 8.00 ETB per kilogram. Based on the CIMMYT^37^ methodology, the treatment involving 110 kg N ha⁻¹ combined with a 30 cm intra-row spacing resulted in the highest net benefit of 236,614.10 ETB ha⁻¹, followed closely by the combination of 110 kg N ha⁻¹ with 20 cm spacing. In contrast, the lowest net benefit was recorded for the control treatment (no nitrogen) at 20 cm intra-row spacing (Table 8).

In terms of marginal rate of return (MRR), the combination of 110 kg N ha⁻¹ and 30 cm spacing also recorded the highest return at 236,614.10%, indicating a highly profitable input investment. This was followed by the treatment with 110 kg N ha⁻¹ and 40 cm spacing, which also showed a favourable return (Table 9). The very high MRR values are mainly due to the small difference in total variable costs between successive non-dominated treatments compared with the substantial increase in net benefits. Increasing nitrogen rate and adjusting plant spacing required only a modest additional investment, while the yield improvement generated a disproportionately higher return. Because MRR is calculated as the change in net benefit divided by the change in cost, even a small cost increase with a large net benefit gain result in a very high MRR.

**Table 8:** Variable cost and gross incomes of potato as influenced by N fertilize and Intra-row spacing.

*N* fertilizer	Intra-row spacing	Seed cost (Eth-Birr)	Fertilizer cost	Labor cost	TVC	MTY (t ha^− 1^)	AdY (t ha^− 1^)	Gross income (Eth-Birr)	Net benefit (Eth-Birr)
0	20	71,250	0	0	71250.0	15.46	13.91	111,312	40062.00
	30	47499.9	0	0	47499.9	17.76	15.98	127,872	80372.10
	40	37999.95	0	0	37999.95	18.59	16.73	133,848	95848.05
55	20	71,250	715	400	72365.0	19.91	17.92	143,352	70987.00
	30	47499.9	715	400	48614.9	19.96	17.96	143,712	95097.10
	40	37999.95	715	400	39114.95	23.18	20.86	166,896	127781.05
110	20	71,250	1430	800	73480.0	41.38	37.24	297,936	224456.00
	30	47499.9	1430	800	49729.9	39.77	35.79	286,344	236614.10
	40	37999.95	1430	800	40229.95	35.25	31.73	253,800	213570.05
165	20	71,250	2145	1200	74595.0	28.16	25.34	202,752	128157.00
	30	47499.9	2145	1200	50844.9	31.1	27.99	223,920	173075.10
	40	37999.95	2145	1200	41344.95	31.88	28.69	229,536	188191.05

Seed price of Belete variety = 15.00 Eth-Birr kg^− 1^, price of N fertilizer = 13 Eth-Birr kg^− 1^; price of potato at farm gate = 8.0 Birr kg^− 1^; labor cost = 100 Eth-Birr per man per day; TVC = total variable cost; MTY = marketable tuber yield; AdY = Adjusted Yield.

**Table 9:** Marginal rate of return of potato as influenced by N fertilizer and intra row spacing.

Treatment combinations	Total variable cost (Birr ha^− 1^)	Net benefit (Birr ha^− 1^)	Dominance Analysis	MRR (%)	Rank
0 × 40	37999.95	95848.05			
55 × 40	39114.95	127781.05		2863.95	3
110 × 40	40229.95	213570.05		7694.08	2
165 × 40	41344.95	188191.05	DO		
0 × 30	47499.90	80372.10	DO		
55 × 30	48614.90	95097.10	DO		
110 × 30	49729.90	236614.10		12692.11	1
165 × 30	50844.90	173075.10	DO		
0 × 20	71250.00	40062.00	DO		
55 × 20	72365.00	70987.00	DO		
110 × 20	73480.00	224456.00	DO		
165 × 20	74595.00	128157.00	DO		

Notes: DO = Dominated; MRR = marginal rate of return.

## Conclusion and recommendation

The results of the present study clearly demonstrated that nitrogen fertilizer and intra-row spacing significantly influenced key growth and yield parameters of potato, including days to flowering, days to maturity, and most other measured traits. In addition, the interaction between N fertilizer and intra-row spacing significantly affected 50% flowering, stem number, yields of very small, small, and large-sized tubers, average tuber weight, marketable tuber yield, and total tuber yield. The highest marketable tuber yield (41.38 t ha⁻¹) was obtained from the treatment combining 110 kg N ha⁻¹ with a 20 cm intra-row spacing. However, based on the analysis of the MRR, the combination of 110 kg N ha⁻¹ with 30 cm spacing was found to be the most economically viable for seed potato production of the Belete variety, recording the highest MRR (12,692.11%). Therefore, the use of 110 kg N ha⁻¹ combined with a 30 cm intra-row spacing is recommended for profitable and efficient seed potato production in the study area and other locations with similar agro-ecological conditions. However, it is important to note that this experiment was conducted at a single location and during one growing season. Therefore, to strengthen and validate these findings, further studies across multiple locations and seasons are recommended to account for potential environmental variability, genotype × environment (G×E) interactions, and seasonal fluctuations that may influence the conclusions drawn from a single-site study. In addition, a brief consideration of the potential environmental impacts associated with nitrogen (N) fertilizer use—such as nutrient leaching, greenhouse gas emissions, and soil degradation would further strengthen the practical recommendations and support more sustainable nutrient management strategies.

## Data Availability

The datasets used and/or analysed during the current study are available from the corresponding author on reasonable request.
